# Impact of the COVID-19 pandemic on the spontaneous reporting and signal detection of adverse drug events

**DOI:** 10.1038/s41598-023-46275-w

**Published:** 2023-11-01

**Authors:** Diana Montes-Grajales, Ricard Garcia-Serna, Jordi Mestres

**Affiliations:** 1 Chemotargets SL, Parc Científic de Barcelona, Baldiri Reixac 4 (TR-03), 08028 Barcelona, Catalonia Spain; 2 Institut de Quimica Computacional i Catalisi, Facultat de Ciencies, Universitat de Girona, Maria Aurelia Capmany 69, 17003 Girona, Catalonia Spain

**Keywords:** Drug discovery, Drug safety

## Abstract

External factors severely affecting in a short period of time the spontaneous reporting of adverse events (AEs) can significantly impact drug safety signal detection. Coronavirus disease 2019 (COVID-19) represented an enormous challenge for health systems, with over 767 million cases and massive vaccination campaigns involving over 70% of the worldwide population. This study investigates the potential masking effect on certain AEs caused by the substantial increase in reports solely related to COVID-19 vaccines within various spontaneous reporting systems (SRSs). Three SRSs were used to monitor AEs reporting before and during the pandemic, namely, the World Health Organisation (WHO) global individual case safety reports database (VigiBase®), the United States Food and Drug Administration Adverse Event Reporting System (FAERS) and the Japanese Adverse Drug Event Report database (JADER). Findings revealed a sudden over-reporting of 35 AEs (≥ 200%) during the pandemic, with an increment of the RRF value in 2021 of at least double the RRF reported in 2020. This translates into a substantial reduction in signals of disproportionate reporting (SDR) due to the massive inclusion of COVID-19 vaccine reports. To mitigate the masking effect of COVID-19 vaccines in post-marketing SRS analyses, we recommend utilizing COVID-19-corrected versions for a more accurate assessment.

## Introduction

Coronavirus disease 2019 (COVID-19) is an infection caused by a positive-sense single-stranded RNA virus, called Severe Acute Respiratory Syndrome Coronavirus 2 (SARS-CoV-2)^1^. Signs and symptoms of this disease include cough, fever, myalgia, weakness, respiratory distress, chills, smell and taste disorders^2^ as well as a broad range of clinical manifestations associated with the cardiovascular, neurological and gastrointestinal systems^3^. The effects of SARS-CoV-2 are highly variable, ranging from asymptomatic infections and mild effects to severe illness with the requirement of hospitalisation and ventilatory support^4^. In addition, over fifty long‑term sequelae of COVID-19 have been identified through systematic review and meta-analysis, which include fatigue, headache, attention disorder, dyspnea and hair loss^5^.

COVID-19 pandemic has affected more than 200 countries and territories^6^. The first COVID-19 patient admission occurred on 12/Dec/2019, in China, followed by a cluster of cases in Asia. Around mid-January, the virus started to spread to other continents^6^. The first case in the United States was reported on 20/Jan/2020^7^ and two months later, on 11/Mar/2020, the pandemic was declared by the World Health Organisation (WHO). Since then, several strategies were implemented to mitigate the effects of the pandemic, including massive vaccination campaigns, lockdowns, and social distancing^8^. Reference^8^ until 6/Oct/2023, over 771 million cases and more than 6.9 million deaths worldwide have been confirmed by the WHO and over 13 billion vaccine doses have been administered^9,10^. This global health issue has had an impact on the utilisation of healthcare systems with a significant reduction of minor ailment consultations^11^ and massive vaccination campaigns against COVID-19^12^, which may have affected the number and type of adverse events (AEs) deposited in spontaneous reporting systems (SRSs) during the pandemic period^13,14^.

Disproportionality measures, such as the proportional reporting ratio (PRR), play a key role in pharmacovigilance to identify signals of disproportionate reporting (SDR) by evaluating the frequency of a specific adverse event associated with a particular drug relative to all other adverse events reported for that drug^15,16^. The chi-square test, a widely used statistical method, helps assess the significance of the observed differences in adverse event reporting, aiding in the detection of potential associations between drugs and adverse effects^16^. Furthermore, comprehending and effectively addressing the masking effect (where a significant adverse event might eclipse the reporting of less frequent but equally significant adverse events) is crucial for comprehensive pharmacovigilance analysis^17–19^, especially after COVID-19 pandemic. While there is no universally accepted standard methodology^20^, several studies have applied these approaches to analyse adverse event reporting trends and safety signals impacted by the COVID-19 pandemic across distinct databases^21^ or within smaller datasets with a limited number of AEs under study^12^.

Understanding the impact of the COVID-19 pandemic on the reporting of AEs in SRSs is crucial to unravel variations that can affect pharmacovigilance analyses^13^, especially due to the sensitivity of some algorithms for quantitative signal detection that use reporting disproportionality to establish causal relationships between drugs and adverse effects ^22,23^. To this end, this work aims at studying the masking effect of COVID-19 vaccines on the WHO global individual case safety reports database (VigiBase®), the United States Food and Drug Administration Adverse Event Reporting System (FAERS), and the Japanese Adverse Drug Event Report database (JADER) through an analysis of the reporting trends and SDR before, during and after the pandemic peak period.

## Methods

### Data sources

Individual case safety reports (ICSRs) were retrieved from VigiBase® Extract Case Level^24^, FAERS^25^, and JADER (PMDA)^26^ databases (last updated on 30/Jan/2023). On the other hand, historical data of COVID-19 cases and deaths, as well as vaccination trends until 31/Dec/2021, were downloaded from the Our World in Data (OWID) COVID-19 repository^9^, which includes information provided by the government of several countries and the data from WHO (last accessed on 30/Jan/2023). Epidemiological information was structured in periods of semesters by summing available daily data (location: “world”) up to 31/Dec/2021. All methods were carried out in accordance with the relevant guidelines and regulations.

### Data preparation

Inter- and intra-database duplicates were removed from VigiBase®, FAERS and JADER as they can affect the outcomes of post-marketing analyses^27^. Duplicate detection was based on field matching of “sex”, “region”, “age”,” drug codes”, “indication”, “adverse event”, “time to onset mean”, “basis” and “event day”, with a previous transformation step to make fields comparable in terms of units or ranges. Three types of duplicates were considered according to the degree of coincidence: (a) reports with exact matches in all the compared fields; (b) reports that miss one element in only one of the fields “adverse events”, “basis” or “indications”; and (c) reports that exhibit a change in a single character in only one of those three fields (Levenshtein distance = 1), with exact matches in the rest of the nine compared fields. In the process of removing inter-database duplicates, the Vaccine Adverse Event Reporting System (VAERS)^28^ was exclusively utilized to identify and eliminate duplicates between databases, as reports of COVID-19 vaccines were taken as a reference background. Entries present in both the specified databases (FAERS, JADER, and VigiBase) and VAERS were eliminated. VAERS was not included in the selected SRSs, and consequently, no analysis of SDR was conducted for this database.

Only reports containing AEs correctly mapped to Medical Dictionary for Regulatory Activities (MedDRA version 23.1) codes and registered until 31/Dec/2021 were considered for further analysis. Report dates and identifiers, along with AEs (MedDRA codes, terms, and systems), were obtained using custom SQL queries in Python 3.8.10. AEs were filtered to remove AE-drug relationships of known indications suspiciously being reported as AEs and terms that do not represent AEs. Additionally, the resultant list of AEs from VigiBase®, FAERS and JADER was manually curated to further detect terms not related to possible AEs. This process identified a total of 17,530 MedDRA preferred terms associated with AEs. In order to focus on the most reported AEs, only MedDRA codes corresponding to AEs with a total number of report count (up to 31/Dec/2021) of 10,000 or more were included in the analysis. This resulted in a list of 919 AEs that met this criterion, as presented in Supplementary Tables S1, S2. The selected AEs account for 5.24% of the total number of AEs in these SRSs.

### Data segmentation

To facilitate the temporal analysis, the number of reports per MedDRA code were organised in annual periods (from 2017–2021) and, for the years between 2019 and 2021, in six semi-annual periods covering two pre-pandemic semesters (from 1/Jan/2019 to 31/Dec/2019) and four pandemic semesters (from 1/Jan/2020 to 31/Dec/2021). Furthermore, reports solely registering COVID-19 vaccines (without other medicinal products) were identified by ingredient codes and removed to create counterpart datasets. These were utilised for comparison purposes with the datasets containing all accepted reports for VigiBase®, FAERS and JADER.

The semi-annual number of COVID-19 cases, deaths and vaccines administered from 1/Jan/2019 to 31/Dec/2021 were obtained from historical data available in the OWID COVID-19 dataset^9^. These data, collected in Table 1, show a steady increase in the number of cases, deaths and vaccines administered from the first semester of 2020 to the second semester of 2021. Only the number of deaths decreased in the second semester of 2021.

**Table 1:** Number of coronavirus disease 2019 (COVID-19) cases, deaths and vaccines administered during four semesters of the COVID-19 pandemic, from 1 January 2020 to 31 December 2021, denoted as pandemic periods 1–4, respectively.

Period	Dates	Number of cases	Number of deaths	Number of vaccines administered
Pre-pandemic 1 (1st semester 2019)	1/Jan/2019–30/Jun/2019	0	0	0
Pre-pandemic 2 (2nd semester 2019)	1/Jul/2019–31/Dec/2019	0	0	0
Pandemic 1 (1st semester 2020)	1/Jan/2020–30/Jun/2020	1,0474,820	552,096	0
Pandemic 2 (2nd semester 2020)	1/Jul/2020–31/Dec/2020	73,300,794	1,349,619	11,906,791
Pandemic 3 (1st semester 2021)	1/Jan/2021–30/Jun/2021	98,932,775	2,074,986	3,102,904,910
Pandemic 4 (2nd semester 2021)	1/Jul/2021–31/Dec/2021	105,992,117	1,492,411	6,063,042,563

For the sake of completeness, the two semesters prior to the COVID-19 pandemic are also included, from 1 January 2019 to 31 December 2019, denoted as pre-pandemic periods 1 and 2.

### Statistical analyses

#### Percentage change of annual RRF 

Trends in the relative reporting frequency (RRF) associated with AEs were analysed by calculating the percentage change in the yearly time series of RRF from 2017 to 2021. All ICSRs registered in this 5-year period were utilized for the analysis. This involved employing SQL custom queries to retrieve the number of unique reports per AE in each period and the number of reports up to 31/Dec/2021. This information, along with the total counts of distinct reports per year (between 2017 and 2021) or semi-annual period (between 1/Jan/2019 and 31/Dec/2021) considering all AEs, were utilised to calculate the RRF of each AE per year or period. To do that, the counts of distinct reports for a specific AE and period were divided by the total number of unique reports registered in the same period.

The change in the RRF for each AE in comparison with the previous year (between 2017 and 2021) was calculated by using the percentage change^29^ *“pct_change()”* function in Python 3.8.10. Percentage change is defined as the subtraction of the initial value from the final value, all divided by the initial value. The results are multiplied by 100 to convert them to percentages (%). In order to limit the number of AEs included in our analysis, only AEs with at least one report between 2017 and 2021 that presented a total number of reports (31/Dec/ 2021) equal or higher than 10,000 were considered for this analysis. Percentage change analysis was carried out by using the RRF per year of each AE in VigiBase®, FAERS and JADER individually but also for the resulting integrated database with intra- and inter-duplicate reports removed and the counterpart datasets with reports exclusively registering COVID-19 vaccines eliminated. Descriptive statistics was carried out to obtain an overview of the annual percentage changes presented for these datasets by using the “*describe*” function of Pandas in Python 3.8.10.

#### Sankey graph and heatmap

AEs were ranked by the percentage change of RRFs obtained between the years 2020 and 2021. The top-25 AEs of the resultant list were used to generate a Sankey diagram and a heatmap by making use of the “*plotly.graph_objects.sankey*” package and the “*seaborn*” library in Python 3.8.10., respectively. This Sankey diagram illustrates the percentage change of RRFs between 2020 and 2021 for each of the top-25 AEs. An extended version of the Sankey diagram was generated for the supplementary information with additional features. The extended version shows the flow in RRF values of these AEs between the years 2017 and 2021, ranked according to the RRF values for each year. On the other hand, the annual number of reports for each AE between 2017 and 2021 was divided by the total number of reports published for the same AE (up to December 31st, 2021) and multiplied by 100. These percentages were used to elaborate a heatmap showing the portion of the total number of reports published in each year between 2017 and 2021.

#### Changes in SDR detection

The entire sample of 919 AEs was utilized to study changes in the number of drugs having those AEs disproportionally reported. The PRR was calculated by using three datasets: i) all unique reports registered before the COVID-19 pandemic (up to 31/Dec/2019), ii) all unique reports registered up to 31/Dec/2021, and iii) all unique reports registered up to 31/Dec/2021 with reports solely registering COVID-19 vaccines excluded. On the other hand, SDR were determined by using the following thresholds: proportional reporting ratio (PRR) ≥ 2, Chi square ≥ 4 and number of individual cases ≥ 3^12,16^ within each individual SRS and dataset^12^. The masking ratio (MR) was calculated by dividing the PRR of a specific drug-AE pair excluding COVID-19 reports by the PRR of the drug-AE pair considering all reports^30^. Similarly, the MR of the lower bound of the 95% confidence interval (MRCI) was calculated by using PRR05 instead of PRR. The average of the MR and MRCI was calculated. Furthermore, the data of the 25 most affected AEs was used for comparative purposes with the whole sample of 919 AEs. Bar plots and boxplots were created in Python 3.8.10. (by using pandas and matplotlib libraries) to visualize the fluctuations in the number of SDR obtained from ICSRs deposited up to 31/Dec/2021 (with and without reports solely registering COVID-19 vaccines), in comparison with those obtained before the COVID-19 pandemic (up to 31/Dec/2019).

## Results and discussion

The total number of reports in VigiBase®, FAERS and JADER registered annually from 2017 to 2021, both including all entries (NRtotal) and excluding reports solely related to COVID-19 vaccines (NRwoVAC), are presented in Table 2. Among the total number of integrated unique reports deposited up to 31/Dec/2021 (32,206,604), 1,485,225 reports (4.6%) are related solely to COVID-19 vaccines. Most of them were deposited in 2021 (99.7%) and come from VigiBase® (98.8%). In this respect, the relative impact of the COVID-19 pandemic on the three SRSs is clear: while FAERS is mostly unaffected, almost half (49.2%) of the reports deposited in VigiBase® in 2021 come from COVID-19 vaccines.

**Table 2:** Total number of unique annual reports (NRtotal) and the number of reports after excluding all reports containing solely coronavirus disease 2019 (COVID-19) vaccines (NRwoVAC) in VigiBase®, Food and Drug Administration Adverse Event Reporting System (FAERS) and Japanese Adverse Drug Event Report database (JADER) in the 5-year period comprised between 2017 and 2021.

Year	VigiBase®		FAERS		JADER	
	NRtotal	NRwoVAC	NRtotal	NRwoVAC	NRtotal	NRwoVAC
2017	1,357,005	1,357,005	1,021,985	1,021,985	54,181	53,985
2018	1,179,527	1,179,527	1,126,467	1,126,467	57,559	57,304
2019	1,322,441	1,322,441	1,151,674	1,151,670	56,796	56,603
2020	1,978,356	1,977,547	1,086,215	1,086,202	46,107	45,932
2021	2,982,785	1,514,748	1,038,473	1,038,293	74,329	61,305
Total	16,301,971	14,833,125	15,188,324	15,188,124	716,309	700,130

As reference, the total number of unique reports (up to 31/Dec/2021) in the integrated spontaneous reporting systems (SRSs) are also provided (last row).

The number of reports and corresponding RRF values, per year and per semi-annual period, with inclusion or exclusion of reports solely related to COVID-19 vaccines, for each of the 919 individual AEs are provided in the electronic supplementary material (Supplementary Tables S1–S8). Descriptive statistics of the mean absolute values of the annual percentage change in the RRF of those 919 AEs within the five-year period between 2017 and 2021 are provided in Table 3 (Supplementary Table S9 includes the whole RRF percentage change data). As can be observed, between 2017 and 2020 the annual percentage changes of RRF remained stable within the range of 12.87 to 19.50% and those values were obviously unaffected during this period upon exclusion of reports considering COVID-19 vaccines only. In contrast, between 2020 and 2021 the mean percentage change of RRFs suddenly went up to 53.25%. However, the value returned to the levels close to those observed during the 2017–2020 period (23.10%) when reports uniquely registering COVID-19 vaccines (without other medicinal products) were excluded. A similar behaviour was obtained for the other statistical measures.

**Table 3:** Descriptive statistics of the absolute value of percentage changes in the relative reporting frequency (RRF) per adverse event (AE) between 2017 and 2021 considering the total number of unique reports and the number of reports after excluding all reports containing solely coronavirus disease 2019 (COVID-19) vaccines (in parenthesis).

Statistical measures	Annual percentage change of RRF values over 919 AEs, includes all entries (excludes reports solely reporting COVID-19 vaccines)			
	2017–2018	2018–2019	2019–2020	2020–2021
Mean (%)	17.79 (17.79)	12.87 (12.87)	19.50 (19.50)	53.25 (23.10)
Std	20.07 (20.07)	17.51 (17.51)	30.44 (30.44)	138.73 (50.46)
Min	0.04 (0.11)	0.01 (0.01)	0.01 (0.00)	0.05 (0.01)
25%	6.59 (6.59)	4.08 (4.08)	7.80 (7.84)	20.02 (4.94)
50%	13.60 (13.60)	8.88 (8.93)	15.92 (16.00)	31.48 (11.74)
75%	22.21 (22.21)	15.08 (15.10)	24.26 (24.26)	41.66 (23.48)
Max	257.8 (257.81)	203.71 (203.70)	498.04 (498.18)	2143.79 (891.96)

Focusing on the absolute value of percentage changes in the RRF per AE for each individual SRS considered in this study (Supplementary Table S10), one observes that between 2020 and 2021 JADER and VigiBase® experienced important increases in those values (233.3 and 74.1%, respectively) compared to the corresponding values in the period between 2019 and 2020 (48.7 and 24.4%, respectively). Removal of reports solely related to COVID-19 vaccines for the 2020–2021 two-year period, largely reduced the average absolute percentage of RRFs in those two SRSs (95.6% and 37.6%, respectively). In contrast, FAERS was mostly unaffected between 2020 and 2021 (18.81%) compared to the previous annual period (18.76%). These results expose the nearly complete absence of reports solely related to COVID-19 vaccines in FAERS due to the FDA's strategic establishment of VAERS as a distinct database specifically dedicated to vaccine-related reports in 1990.

The impact of COVID-19 on the various SRSs is complex and the potential development of some AEs could well be the result of multiple aspects, including drug-drug interactions with COVID-19 treatments and other medicinal products^27^. Table 4 contains the AEs with percentage increase in RRF ≥200 between 2020 and 2021 (see Supplementary Table S9 for the full list of AEs), along with the number of SDR detected by including all reports or with exclusion of reports solely related to COVID-19, the percentage of SDR masked when considering all reports and the average MR (the average MRCI values were not presented as these where almost identical to MR). Overall, all unique reports received in 2021 involving the top-25 highly impacted AEs represent 30.3% of the total number of distinct reports deposited in 2021 in VigiBase®, FAERS and JADER.

**Table 4:** List of 35 adverse events (AE) with relative reporting frequency (RRF) percentage changes ≥ 200% between 2020 and 2021; with the number of signals of disproportionate reporting (SDR) identified by using the total number of unique reports (NRTotal) up to 31/Dec/2021 and by excluding reports solely registering COVID-19 vaccines (NRwoVAC) for VigiBase, FAERS and JADER and the percentage of signals masked by COVID-19 vaccines when considering all reports.

AE	% change of the RRF between, 2020–2021	Number of drugs with disproportionally reported event (PRR ≥ 2, Chi square ≥ 4, and number of individual cases ≥ 3)		Percentage of SDRs masked when considering all reports	Average masking ratio (MR)
		NRtotal	NRwoVAC		
Axillary pain	2143.8	121	218	44.5	2.3
Vaccination site pain	1980.1	137	242	43.4	3.7
Polymenorrhoea	1474.3	40	101	60.4	2.6
Menstrual disorder	1101.0	108	172	37.2	3.5
Extensive swelling of vaccinated limb	958.9	148	163	9.2	1.5
Lymphadenopathy	937.8	258	433	40.4	1.6
Injection site inflammation	910.1	188	215	12.6	1.2
Menstruation delayed	785.5	42	69	39.1	2.0
Vaccination site reaction	747.1	132	169	21.9	2.7
Myalgia	698.5	193	342	43.6	1.6
Menstruation irregular	506.5	70	89	21.3	1.6
Dysmenorrhoea	501.1	62	98	36.7	1.5
Menorrhagia	445.6	74	100	26.0	1.3
Limb discomfort	393.8	155	330	53.0	1.7
Pericarditis	392.7	246	327	24.8	1.3
Facial paralysis	357.1	257	343	25.1	1.2
Influenza like illness	334.5	184	249	26.1	1.4
Amenorrhoea	324.2	121	138	12.3	1.2
Lethargy	321.0	294	440	33.2	1.4
Myocarditis	318.0	190	280	32.1	1.3
Pyrexia	303.7	447	643	30.5	1.3
Formication	303.2	132	197	33.0	1.3
Headache	295.1	221	387	42.9	1.3
Vaccination site swelling	292.2	166	187	11.2	1.4
Metrorrhagia	278.8	89	99	10.1	1.1
Chills	270.9	283	443	36.1	1.4
Application site pain	263.3	220	294	25.2	2.0
Feeling cold	252.9	229	321	28.7	2.0
Vaccination site erythema	251.0	179	200	10.5	1.5
Fatigue	250.1	315	508	38.0	1.3
Injection site haematoma	240.5	121	145	16.6	1.3
Arthralgia	218.2	284	423	32.9	1.2
Malaise	215.7	249	388	35.8	1.3
Tenderness	200.9	382	416	8.2	1.1
Guillain-Barre syndrome	200.0	264	288	8.3	3.8

Among the AEs that showed a percentage change in the RRF between 2020 and 2021 equal to or greater than 200% (Table 4), Guillain-Barre syndrome exhibited the highest average MR with a value of 3.8, followed by vaccination pain (3.7) and menstrual disorders (3.5). This information provides insight into the masking effect on the PRR values caused by reports exclusively related to COVID-19 vaccines. The average MR values indicate that, for some of the AEs presented in Table 4, the PRR values after the unmasking process (which involves removing reports exclusively related to COVID-19 vaccines) not only doubled but sometimes even exceeded triple the PRR values obtained when considering all reports.

Many of the AEs collected in Table 4 have been previously reported to be related to COVID-19 or COVID-19 vaccines^31–35^. For example, axillary pain is the AE having the maximum percentage change in the RRF (2143.8%) for an AE reported between 2020 to 2021 in all SRSs and vaccination site pain, polymenorrhoea and menstrual disorder all have percentage changes in the RRF over 1000%. Some of the AEs having a high percentage change between the RRF of 2020 and 2021 (Supplementary Table S9) have been identified as AEs commonly registered from COVID-19 vaccines in VigiBase®^36^. Myalgia (698.5%), pyrexia (303.7%), headache (295.1%), chills (270.9%), fatigue (250.1%), arthralgia (218.2%), and malaise (215.7%) are found among them. Similarly, other highly disrupted AEs, such as pericarditis (392.7 %), myocarditis (318.0 %) and Guillain-Barre syndrome (200.0 %), were reported as AEs of special interest according to a multi-database study comprising data of seven European countries^37^. Lymphadenopathy, extensive swelling of vaccinated limb, injection site inflammation, vaccination site reaction, dysmenorrhoea, limb discomfort, facial paralysis, influenza like illness, amenorrhoea, lethargy, and formication are other COVID-19 associated AEs all showing percentage changes in the RRF over 300% between 2020 and 2021.

**Figure 1:**
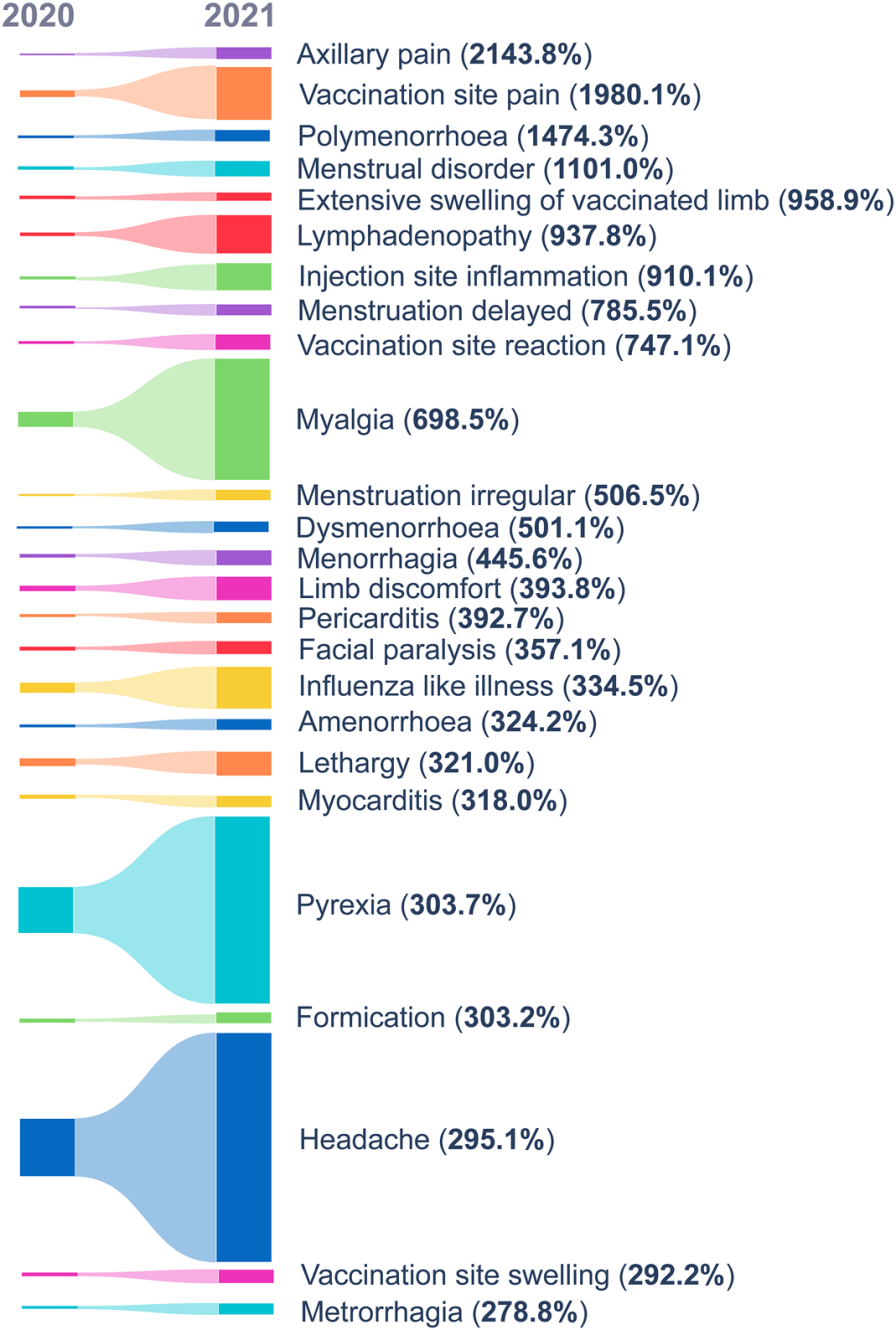
Representative Sankey diagram showing the percentage change in relative reporting frequency (RRF) for 25 adverse events (AEs) significantly impacted by the COVID-19 pandemic between 2020 and 2021. The height of the dark-coloured boxes visually represents the RRF values for both years (To view the full Sankey diagram with RRF values from 2017 to 2021, refer to Supplementary Fig. 1).

To visually strengthen the impact of the COVID-19 period on the reporting of these 25 AEs, a Sankey diagram showing the flow of the RRF during 2020 and 2021 is presented in Fig. 1. Furthermore, we have provided an extended version of the Sankey graph that illustrates the reporting trends for the five-year period spanning from 2017 and 2021 in Supplementary Fig. 1. In both versions, the thickness of the links between nodes and the numbers on the graph represent the RRF in percentages for each AE and year and the use of different colours helps to follow the annual evolution of the RRF for the different AEs. In the extended version, the vertical position of each node represents the relative order of the AEs at each year (labelled on top) according to its RRF.

The extended Sankey graph clearly shows a substantial increase in the RRF of these AEs between 2020 and 2021 compared with the previous three years, as well as the potential of this behaviour to change the order of priority and/or predominance of certain AEs according to the ranking of their annual RRF, possibly masking other relevant AEs not associated with the pandemic. As mentioned above, we can only associate these trends to the addition of an enormous number of reports solely referring to COVID-19 vaccines during the massive immunisation process.

Among the list of the top-25 highly impacted AEs by the massive immunisation process during the COVID-19 pandemic, headache is the AE with the largest RRF in 2021 (14.74%), a value that represents a significant increase over the average RRF in previous years (3.79%) with a percentage change from 2020 to 2021 of 295.1% (Table 4). Pyrexia, myalgia, vaccination site pain, influenza like illness and lymphadenopathy complete the list of six AEs with RRF > 1% in 2021. Of them, vaccination site pain is one of the AEs most impacted by COVID-19 vaccination campaigns. With an average RRF of 0.079% between 2017 and 2020, the RRF in 2021 jumped suddenly to 2.245%, leading to the second largest percentage change (1980.1%) between 2020 and 2021 (Table 4).

**Figure 2:**
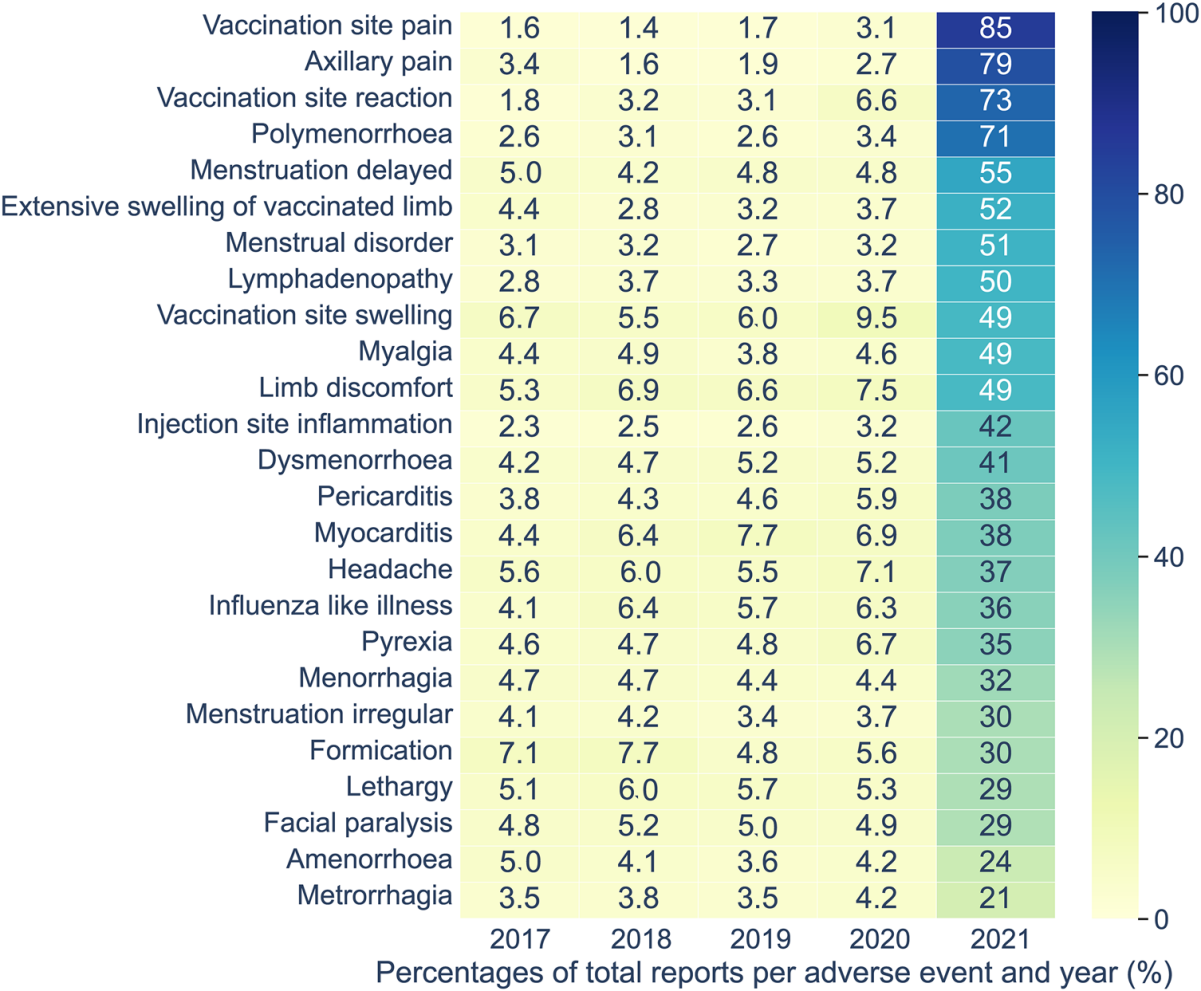
Heatmap representing the percentage of annual reports deposited between 2017 and 2021 relative to the total number of reports for the 25 adverse events (AEs) most impacted by the Coronavirus disease 2019 (COVID-19) pandemic.

Looking at the same reporting data from a longitudinal perspective allows for reassessing the list of 25 AEs most impacted by the COVID-19 pandemic based on the percentage of annual reports deposited in 2021 relative to the total number of reports for each AE (Fig. 2). Vaccination site pain is the AE showing the highest reporting impact in 2021. Of the total number of 107,940 unique reports in which vaccination site pain is mentioned as AE in the different SRSs, 91,966 (85.2%) reports were deposited in 2021 (Supplementary Table S1) and, of those, 80,647 (74.7%) are solely associated with COVID-19 vaccines (Supplementary Table S11). Previously, between 2017 and 2019, the number of vaccination site pain reports would increase at an average annual rate of 1.6%, just to increase slightly to 3.1% in 2020 prior to the huge leap of 85.2% in 2021. Axillary pain, vaccination site reaction, polymenorrhoea, menstruation delayed, extensive swelling of vaccinated limb, menstrual disorder and lymphadenopathy complete the list of AEs having over 50% of their total number of reports deposited in 2021.

**Figure 3:**
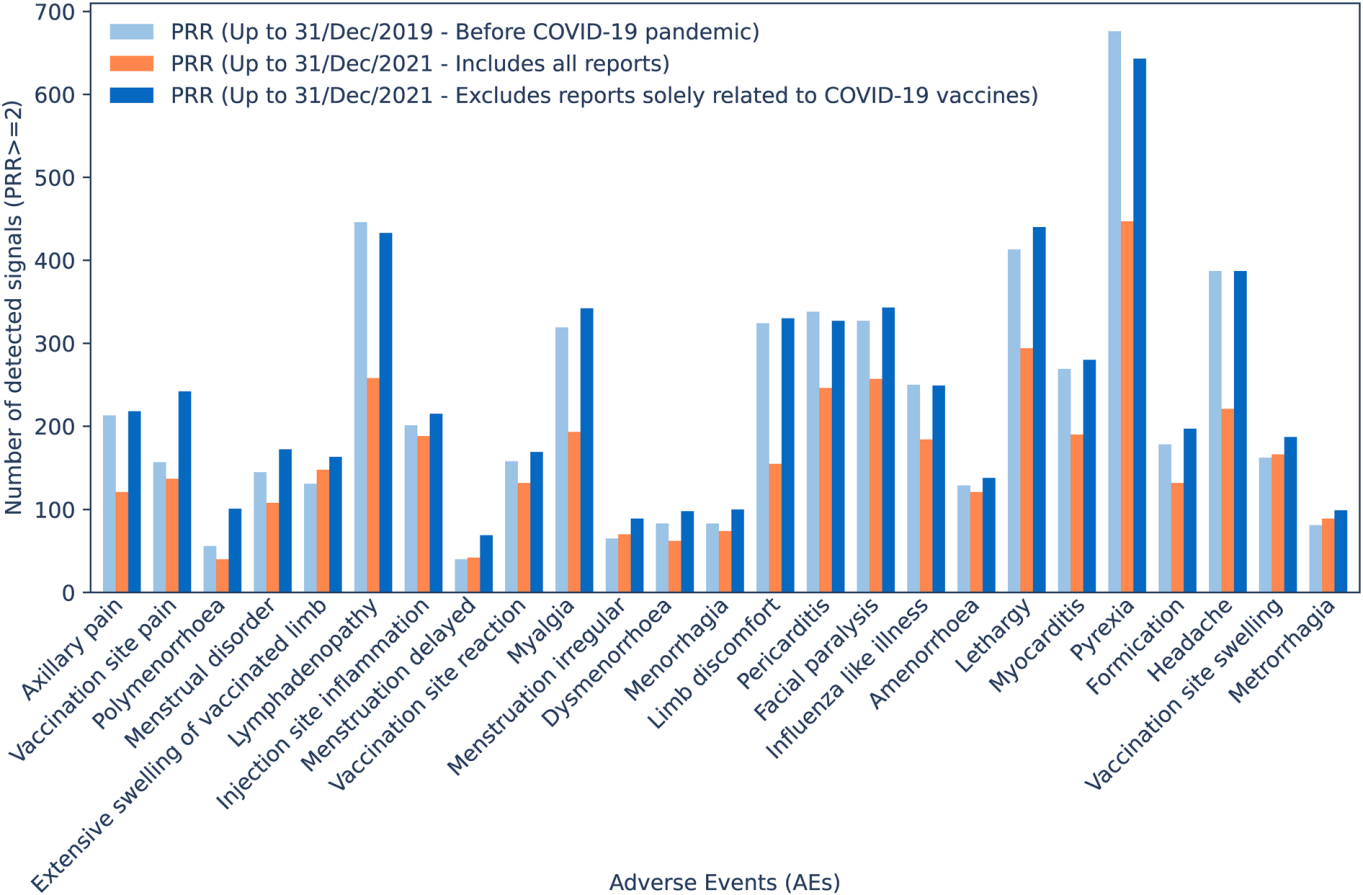
Comparison between the total number of drugs for which the 25 adverse events (AEs) most impacted by the pandemic were disproportionally reported before the Coronavirus disease 2019 (COVID-19) pandemic up to 31/Dec/2019 (light blue) and the corresponding number of drugs using all reports up to 31/Dec/2021 (orange) and excluding entries solely registering COVID-19 vaccines (dark blue).

The abrupt increase in the number of reports highlighted above for certain AEs during the COVID-19 pandemic (Fig. 2) left a permanent mark in VigiBase® and JADER^12^. Such a reporting legacy may potentially have important implications for SDR detection in both integrated and individual SRSs. To quantify such an impact, Fig. 3 illustrates the total number of drugs for which the top-25 AEs highly impacted by the pandemic were disproportionally reported before the COVID-19 pandemic up to 31/Dec/2019 compared with the corresponding number of drugs using all reports up to 31/Dec/2021, on one side, and excluding entries solely registering COVID-19 vaccines, on the other side. As a disproportionality measure, the PRR was used^12^. A side effect was considered disproportionally reported for a given drug if PRR ≥ 2, Chi square ≥ 4, and number of individual cases ≥ 3^12,16^.

There are two general trends that can be extracted upon inspection of Fig. 3. First, consideration of all unique spontaneous reports deposited up to 31/Dec/2021 in SRSs (orange bars) leads to a significant reduction in the number of drugs having any of those 25 AEs disproportionally reported compared to the pre-pandemic situation (light blue bars). And second, when the PRR, Chi square and number of individual cases are recalculated after eliminating all reports solely registering COVID-19 vaccines, then the number of SDR (dark blue bars) returned to the pre-pandemic levels. On average, when comparing the COVID-corrected SRSs (with all COVID-19 vaccine-only reports removed) with the original SRSs (with all reports) there is a 32.4% reduction of SDR on those 25 AEs. In contrast, when comparing the pre-pandemic with the COVID-corrected reports, there is a 7.1% increase of SDR on those 25 AEs, with an 74.6% overlap of the drug-AE pairs detected. Therefore, to reduce the impact of the COVID-19 pandemic on SDR detection, it is important that all SRSs involved in the study are not only deduplicated but all COVID-19 vaccine-exclusive reports are removed prior to performing any disproportionality analyses.

When the same analysis is performed on each individual SRS, one observes that the impact in SDR detection for VigiBase®, FAERS and JADER is significantly different (Supplementary Fig. S1). While FAERS does not lose a single signal across all 25 AEs, VigiBase® loses on average 40% of the SDR. In between, JADER loses on average 29.3% of the SDR for 10 AEs but retains, and even slightly increases in certain cases, the number of SDR for 15 AEs (Supplementary Table S12).

**Figure 4:**
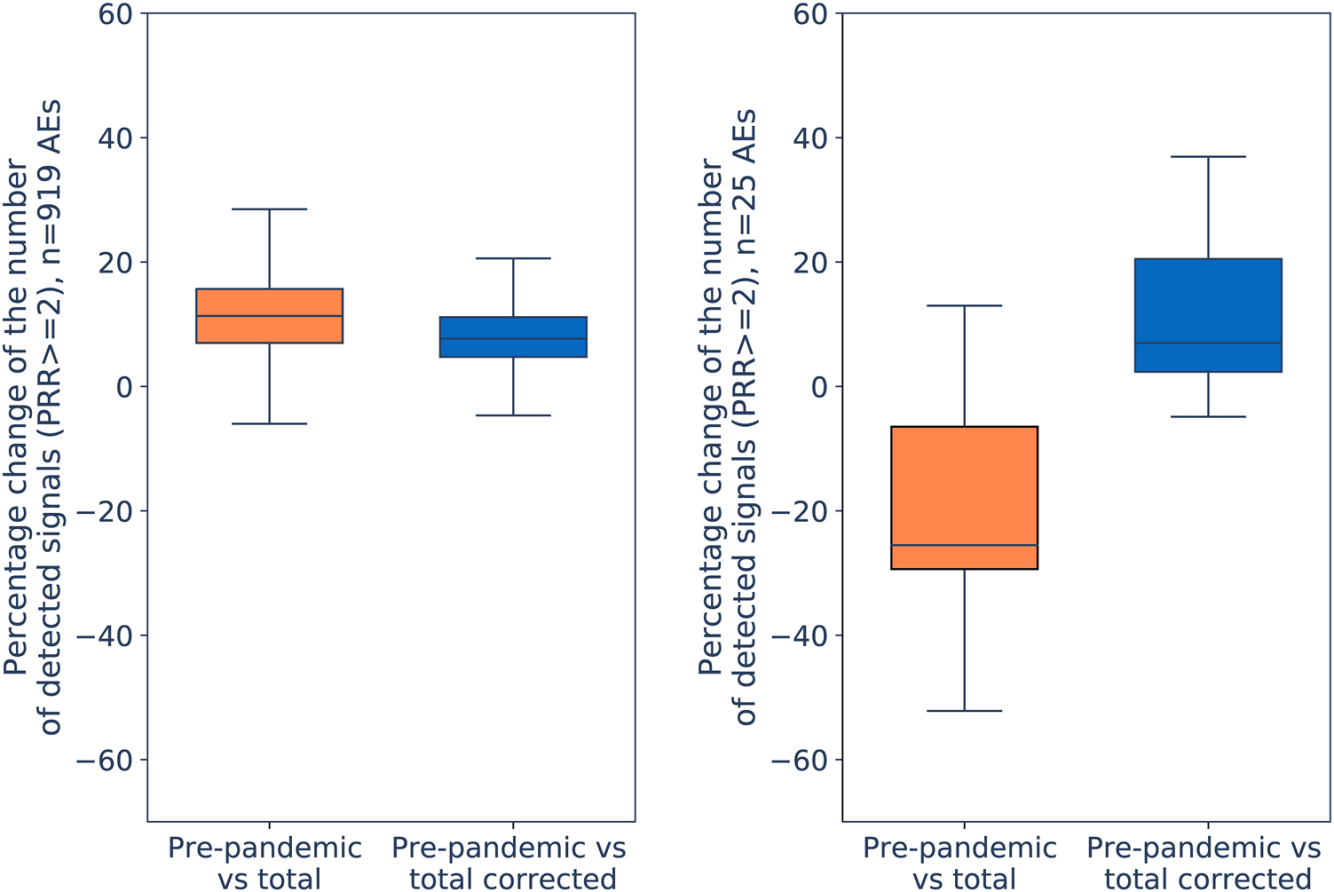
Boxplot distributions of the percentage change between the total number of drugs with disproportionally reported adverse events (AEs) before the Coronavirus disease 2019 (COVID-19) pandemic up to 31/Dec/2019 and up to 31/Dec/2021 by using all reports (orange), on one side, and excluding entries solely registering COVID-19 vaccines (blue), on the other side, for the total number of 919 AEs considered in this study (left) and the 25 AEs most impacted by the COVID-19 pandemic (right).

Finally, we compared the impact of the COVID-19 pandemic on SDR between those highly overreported 25 AEs and the full list of 919 AEs included in this study. Figure 4 displays the boxplot distributions of the percentage change between the number of drugs with disproportionally reported AEs before and after the pandemic. When considering all 919 AEs, most of which were not affected by the pandemic, we observe that there is a slight increase in the number of SDR detected (median: 11.3%) and the same trend is retained when all COVID-19 vaccine reports are removed (median: 7.7%) (Fig. 4 left). In contrast, when analysing the 25 most-affected AEs, a significant decrease in the number of SDR is perceived (median: -25.5%) but this is corrected to a slight increase in SDR detection (to the levels observed for the 919 AEs) when the COVID correction of removing all COVID-19 vaccine-only reports are removed (median: 7.0 %) (Fig. 4 right). In this respect, following the trends highlighted above, VigiBase® and JADER are the most affected SRSs by the COVID-19 pandemic (Supplementary Fig. S2) with the loss of 50% or more SDR for 10 and 7 AEs, respectively (Supplementary Table S12). In contrast, FAERS is found unaffected by the pandemic due to the decision to include all COVID-19 vaccine reports in a separate VAERS SRS.

## Conclusions

SRSs are crucial for the early detection of AEs related to drugs in the post-marketing phase. As these repositories do not include the total number of persons exposed to a specific medicinal product that facilitate the calculation of the incidence of AEs related to it, the methodologies to assess the relationship between drugs and AEs are mainly based on disproportionality measures that compare the reporting rate of an AE for an specific drug with the average RRF for the all other medicinal products^38^. Therefore, the atypical RRF of certain AEs observed during the COVID-19 pandemic has the potential to perturb the identification of SDR and affect post-marketing surveillance.

It was found that the overall RRFs of certain AEs were highly impacted during the COVID-19 pandemic, particularly in 2021. That year, a substantial increase in the average percentage change of absolute RRF in comparison with previous years was observed for VigiBase® and JADER, mostly due to the over-reporting of certain AEs that flourished during the massive immunisation process with COVID-19 vaccines. FAERS managed the issue by creating a separate VAERS database for the reports from COVID-19 vaccines.

A direct consequence of the sharp increase in the number of reports for certain AEs during the COVID-19 pandemic is an important reduction in the number of drugs for which those AEs are found disproportionally reported. The construction of COVID-corrected versions of VigiBase® and JADER is strongly recommended to minimise the permanent mark that the pandemic left on these SRSs and return SDR detection to pre-pandemic levels and trends.

### Supplementary Information

Supplementary Figures.

Supplementary Tables.

## Data Availability

FAERS is publicly available on the FDA website (https://www.fda.gov/drugs/drug-approvals-and-databases/fda-adverse-event-reporting-system-faers). In contrast, JADER dataset is accessible on the PMDA website (www.pmda.go.jp). Furthermore, VigiBase, the WHO global database of reported potential side effects of medicinal products, developed and maintained by Uppsala Monitoring Centre is accessible under specific licensing terms. It is essential to emphasize that the data within VigiBase originates from various sources, and the probability of a suspected adverse effect being linked to a specific drug may vary in different cases. The information presented in this article does not necessarily reflect the views or opinions of the UMC or the WHO. Restrictions apply to the availability of these data, which were used under license for the current research, and so they are not publicly available. However, additional information is available from the authors upon reasonable request and with permission from the institution that holds the license. All data generated during this study are available as supplementary material. Custom python scripts and SQL queries developed for the analyses can be provided by the corresponding author upon request.
