# Supplementary material for: Impact of the COVID-19 pandemic on the spontaneous reporting and signal detection of adverse drug events
Source: Sci Rep. 2023 Nov 1;13:18817. doi: 10.1038/s41598-023-46275-w (PMC10620227; doi:10.1038/s41598-023-46275-w)
Supplement: Supplementary file 1 — Supplementary Figures. [file 41598_2023_46275_MOESM1_ESM.docx]

**SUPPLEMENTARY INFORMATION**

**Title:** Impact of the COVID-19 pandemic on the spontaneous reporting of adverse drug events.

**Authors:** Diana Montes-Grajales^a^, Ricard Garcia-Serna, Jordi Mestres^a,b,*^

**Affiliations:** ^a^ Chemotargets SL, Parc Científic de Barcelona, Baldiri Reixac 4 (TI-05A7), 08028 Barcelona, Catalonia, Spain; ^b^ Institut de Quimica Computacional i Catalisi, Facultat de Ciencies, Universitat de Girona, Maria Aurelia Capmany 69, 17003 Girona, Catalonia, Spain

*** Corresponding author:** Jordi Mestres.

**E-mail:** jordi.mestres@chemotargets.com


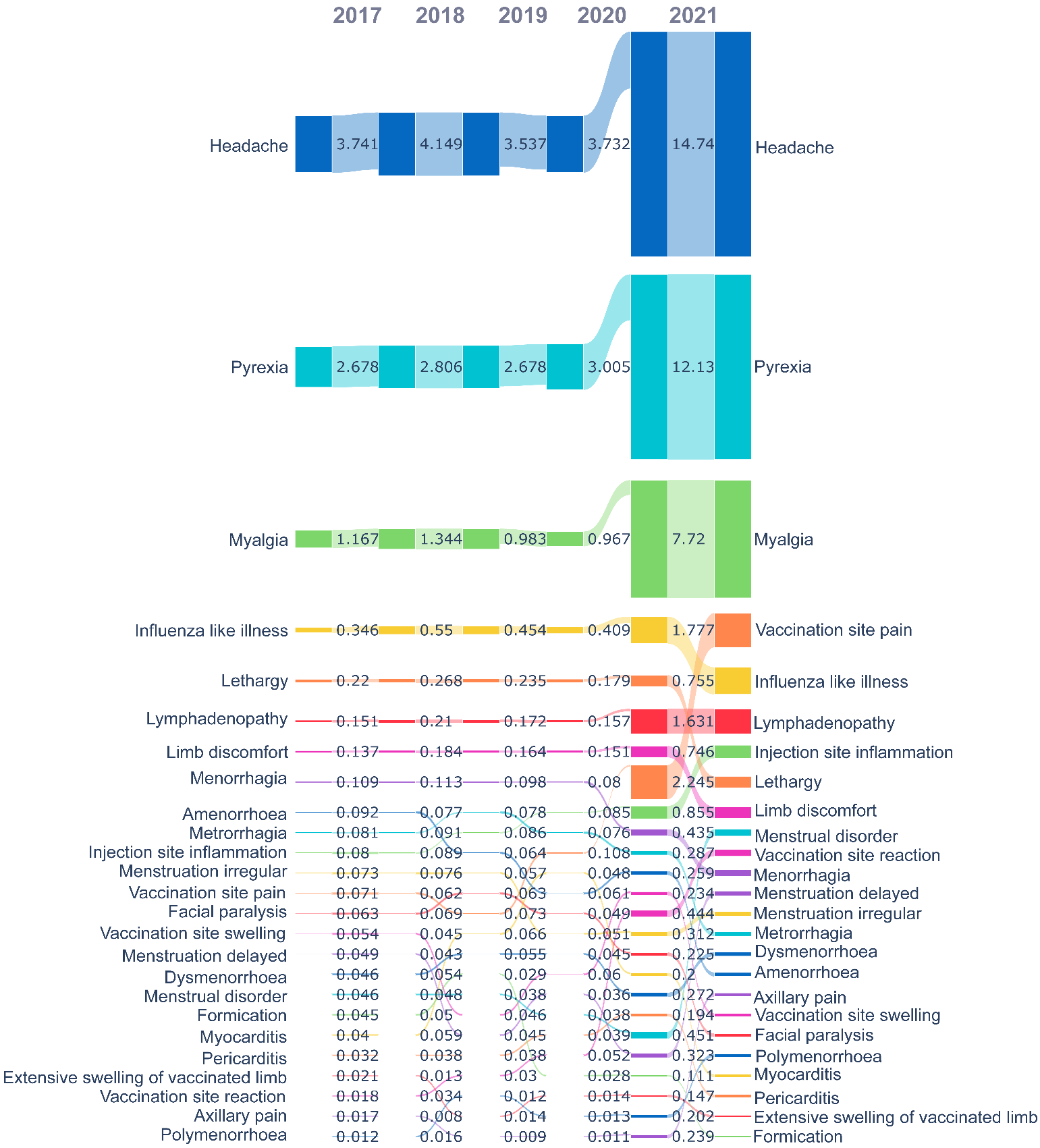


**Supplementary Figure 1.** Sankey diagram showing the flow in the relative reporting frequency (RRF) per year between 2017-2021 for representative adverse events (AEs) highly impacted by the pandemic. The vertical location of the bars represents the relative ranking position of the AEs in terms of RRF at each year (in descending order of RRF, which are shown as numbers -in percentage- on the figure).


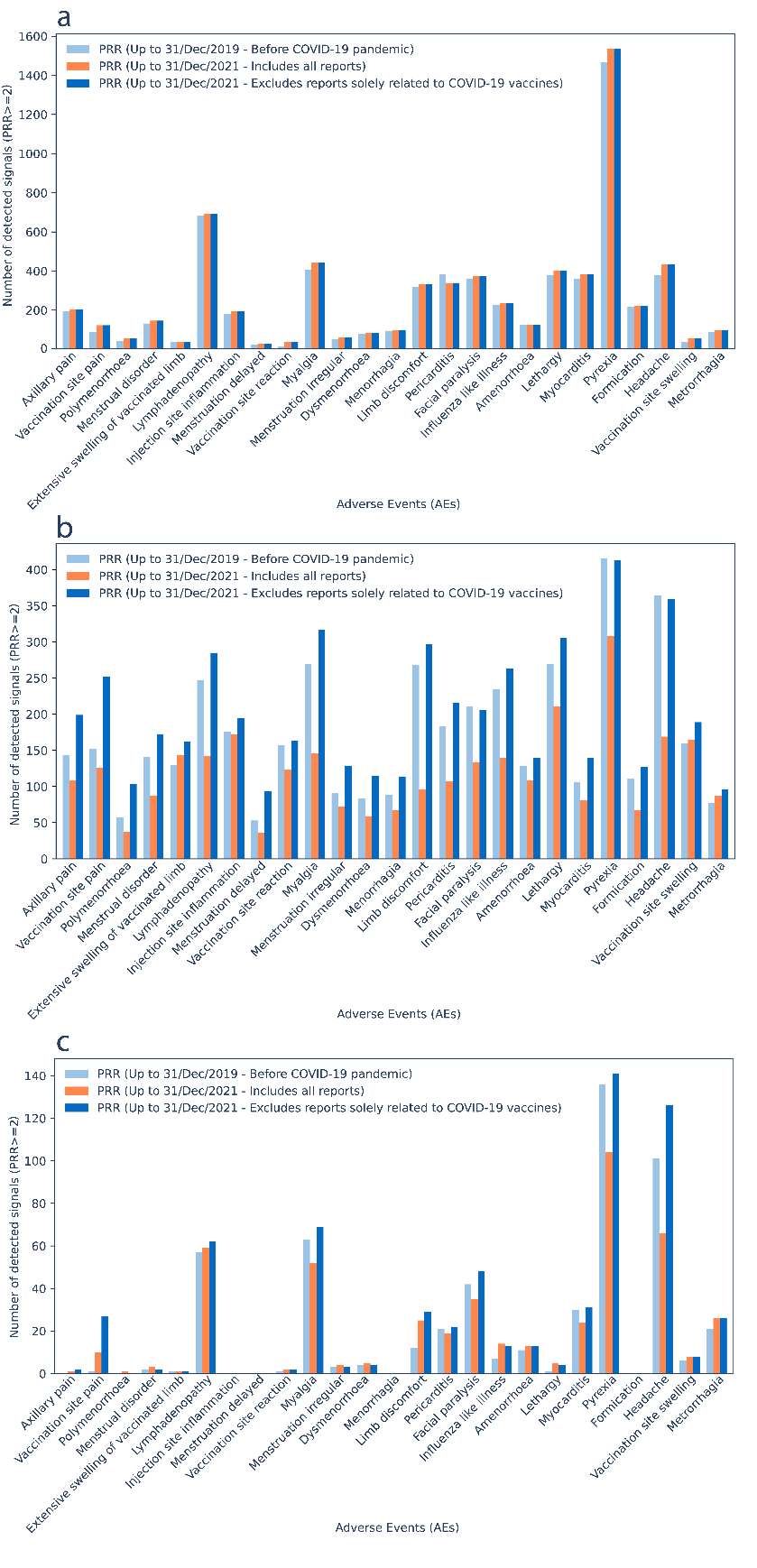


**Supplementary Figure 2.** Comparison between the number of drugs for which the 25 adverse events (AEs) most impacted by the pandemic were disproportionally reported in (a) the Food and Drug Administration adverse event reporting system (FAERS), (b) VigiBase® and (c) the Japanese Adverse Drug Event Report database (JADER), before the *COVID-19* Coronavirus disease 2019 (COVID-19) pandemic up to 31/Dec/2019 (light blue) and the corresponding number of drugs using all reports up to 31/Dec/2021 (orange) and excluding entries solely registering COVID-19 vaccines (dark blue).


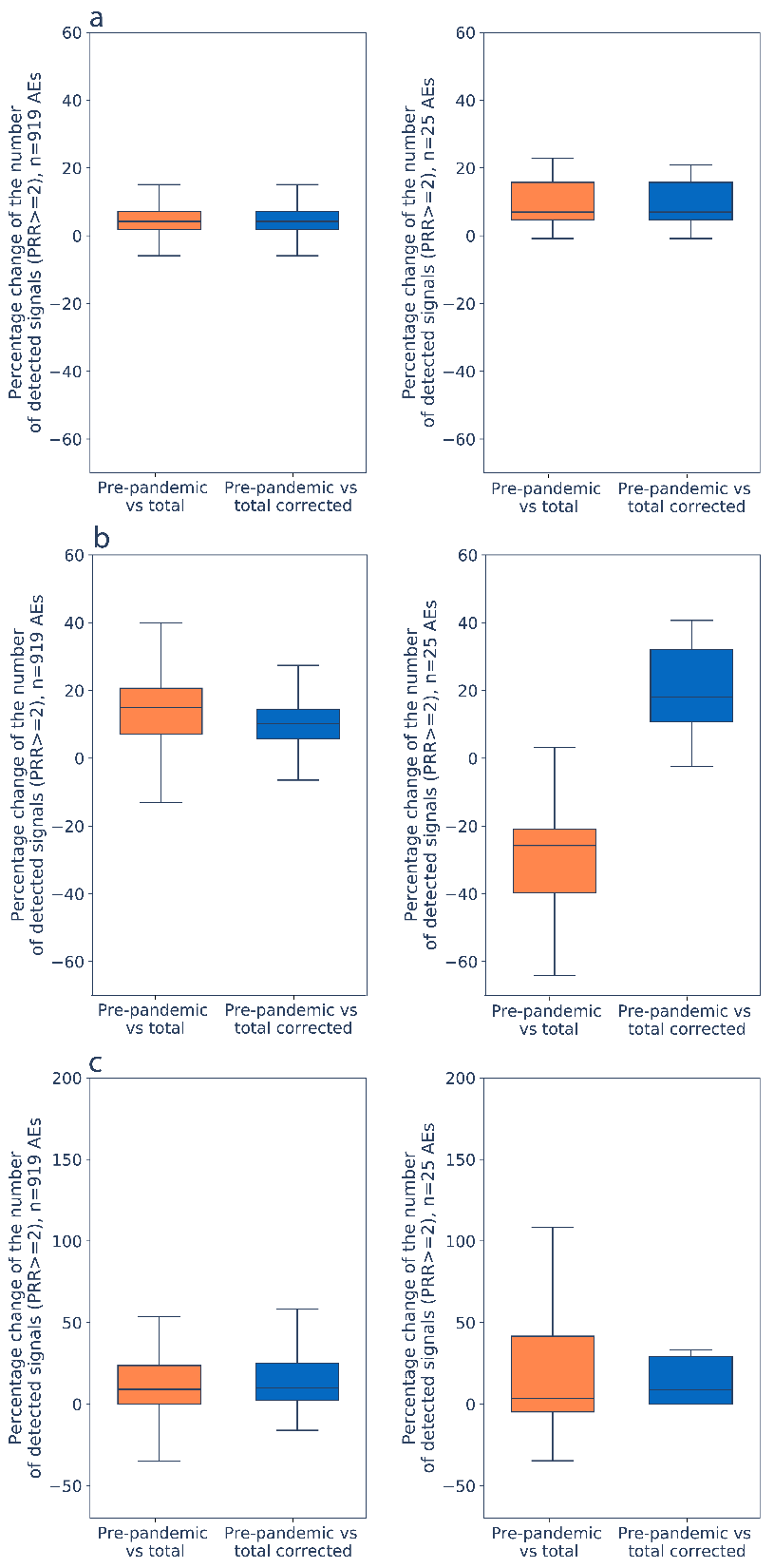


**Supplementary Figure 3.** Boxplot distributions of the percentage change between the number of drugs with disproportionally reported adverse events (AEs) in (a) the Food and Drug Administration adverse event reporting system (FAERS), (b) VigiBase® and (c) the Japanese Adverse Drug Event Report database (JADER), before the *COVID-19* Coronavirus disease 2019 (COVID-19) pandemic up to 31/Dec/2019 and up to 31/Dec/2021 by using all reports (orange), on one side, and excluding entries solely registering COVID-19 vaccines (blue), on the other side, for the total number of 919 AEs considered in this study (left) and the 25 AEs most impacted by the COVID-19 pandemic (right).
